# Comparison of the *Ex Vivo* Expansion of UCB-Derived CD34+ in 3D DBM/MBA Scaffolds with USSC as a Feeder Layer

**Published:** 2013-10

**Authors:** Zahra Sadat Hashemi, Mahdi Forouzandeh Moghadam, Masoud Soleimani

**Affiliations:** 1 Department of Medical Biotechnology, Faculty of Medical Sciences, Tarbiat Modares University, Tehran, Iran; 2 Department of Hematology, Faculty of Medical Sciences, Tarbiat Modares University, Tehran, Iran

**Keywords:** DBM, *Ex vivo* expansion, MBA scaffold, UCB-CD34+, USSC cells

## Abstract

***Objective(s):*** *Ex vivo* expansion of hematopoitic stem cells is an alternative way to increase umbilical cord blood (UCB)-CD34+ cells for bone marrow transplantation. For this purpose demineralized bone matrix (DBM) and mineralized bone allograft (MBA) as two scaffolds based on bone matrix and stem cell niche, were simultaneously used to enhance the effect of human mesenchymal progenitor cells (MPCs) - unrestricted somatic stem cells (USSCs) - as a feeder layer.

***Materials and Methods:*** USSCs were isolated and characterized by morphological and immunological analysis then seeded on both scaffolds as a feeder layer. UCB-CD34^+^ were isolated by MACS method and were co-culture expanded by USSC in 3D and 2D environments. After 3 weeks expansion, cells were counted and were assessed by karyotype, flow cytometry, clonogenic activity, and long-term culture-initiating cells (LTC-IC).

***Results:*** Co-culture expansion in DBM and MBA was 29.22-fold and 27.77-fold, no significant differences in colony and LTC-IC were obtained. Maximum number of colonies belonged to the day 14 with the 73% CFU-GM (Colony Forming Unit- Granulocyte/Macrophage) in contrast to the day 0 which was BFU-E/CFU-E (Burst/Colony Forming Unit-Erythroid). Flow cytometry indicated that the percentage of CD34+ marker was decreased in USSC co-culture and the highest percentage was observed in simple 2D culture.

***Conclusion:*** Because of acid extraction in the DBM production process, mineral materials were removed and the protein background that was more flexible was presented. Therefore these results suggest that USSC-DBM can be a suitable *ex vivo* mimicry niche by intensifying of surface/volume ratio and supporting the stem cell differentiation and expansion.

## Introduction

Currently bone marrow transplantation (BMT) is considered a clinical treatment for many congenital and acquired blood diseases (1). For this treatment hematopoietic progenitor cells (HPCs) are used (2-3). BMT is performed either as an autologous or an allogeneic transplantation. The requirement for hematopoietic stem cells (HSCs) in an allogeneic transplantation is 100,000 times greater than for an autologous transplantation (4). There are three sources for prior HSCs: bone marrow, peripheral blood and cord blood. Between these 3 sources, cord blood sample is the healthy and safe source of HPC cells for the recipient (5, 6). But on the other hand the total number of cells derived from umbilical cord blood (UCB) was low and this is an important barrier. In adult recipients, BMT is limited mainly by a low CD34+ cell dose (7). To overcome this problem, CD34^+^ expansion is recommended (8). Therefore UCB CD34+ cells were *ex vivo* expanded and then injected into the patient to replicate (9). In nature, HSCs are in the microenvironment, or niche, such that they are surrounded by stromal and mesenchymal cells. The niche can control the number of stem cells in the body and prevent them from multiplying too. Interaction between the stem cell and spatial position in the niche establish its equilibrium to proliferation or differentiation (10, 11). Stem cells (SC_s_) cannot survive for long outside the niche (12). *Ex vivo*-like conditions are composed of a three-dimensional (3D) structure (scaffold) and a feeder layer. 

Scaffolding features are similar to the extracellular matrix (ECM). ECM has an important role in physical and chemical signals that are amplified. The 3D environment for HSC niche with helper cells is associated with the basal material. In 3D cultures, cells are grown in a type of *in vivo*-like morphology and the interaction between them can be done better. Three dimensional cultures in tissue engineering research by using biochemical materials as the scaffold is rapidly growing. 

3D structures in tissue engineering is a multidisciplinary field and is a new and exciting technique. It has the potential to create organs and tissues *de novo *(13). It involves the *in vitro seeding* and attachment of human cells onto the scaffold. These cells then proliferate, migrate and differentiate into the specific tissue while secreting the extracellular matrix components required for creating the tissue. These scaffolds have some major properties: They are biocompatible, injectable and biodegradable (14). The 3D structure is used for providing increased surface/volume ratio of culture. These factors let the scaffold improve the efficiency of the culture.

Scaffolds are critical, both *ex vivo* as well as *in vivo*, to mimic the *in vivo* milieu and allow cells to influence their own microenvironments. Here, we try to imitate HSC microenvironment or niche in 3D culture. For bone marrow and cartilage, the most commonly used scaffolds are MBA (mineralized bone allograft) and DBM (de-mineralized bone matrix) (15). Basically, they are used for repair, clinical surgery, bone allograft and transplantation (16). These are based on the bone matrix but the only difference between them is the lack of mineralized materials in DBM by the acid extraction method (17). As described above, to provide a similar environment *in vitro*, human cord blood as a novel source of human mesenchymal progenitor cell (MPC) (18) - unrestricted somatic stem cells (USSCs) - was integrated in an attempt to use as a feeder layer (19-21). USSC cells on the MBA and DBM scaffolds are same as the matrix for UCB-CD34+ cell growth support. The culture system appeared to provide a different microenvironment from the 2D flask culture and might be used as an alternative model for hematopoiesis. 

Our goal then, understands the importance of the presence of the mineral materials for the expansion of HSCs and the extent of the influence of the self-renewal of stem cells in the 3D culture by USSC feeder in contrast to the 2D culture. This research aims to increase the number of hematopoietic stem cells with minimal genetic manipulation in three-dimensional culture. However, to reduce the risk of rejection minimal genetic manipulation and techniques should be involved. 

## Materials and Methods

***Generation and expansion of USSCs from fresh UCB***

UCB samples were collected from full term, normal deliveries according to the approved ethical procedures at Tarbiat Modares University (Iran, Tehran, Shariati Hospital). From each donor, informed consent was acquired according to patient sample collection for research studies protocol approved by the ethics committee. After that USSCs were isolated according to a protocol by Kogler *et al*. To isolate the mononuclear cells (MNCs) and reduce RBC contamination of the upper phase to the minimum level, low-density (less than 1.077 g/ml) cell isolation was performed by Ficoll-Hypaque gradient centrifugation (Pharmacia, Uppsala, Sweden). After 2 washes by phosphate buffered saline (PBS), cells were cultured in T25 flasks and specific media: low-glucose DMEM supplemented with 10^-7^ M Dexamethasone, 30% FCS, 2 mM ultraglutamine, 100U/ml penicillin G, 100 µg/ml streptomycin, it will be better by used of Myelocult medium containing 12.5% FCS and 12.5% horse serum. Cells were incubated at 37°C in 5% CO_2_ in a fully humidified. After two weeks the adherent USSC colonies were observed. 

***Monoclonal antibodies for immunophenotyping of USSCs***

Fluorescein isothiocyanate-conjugated antibodies (Abs) were obtained as follows: USSC cells were analyzed by flow cytometry assay to detect their markers. These cells were positive for CD166, CD73, CD105, CD90, CD44, weak positive expression for KDR, and negative for CD117, CD34, CD45 and Cadherin V.

***Transdifferentiation of USSCs into adipocytes and osteoblasts***

In order to confirm the source USSCs as the human mesenchymal progenitor cells, and the process of differentiation, we check differentiation potential of USSC cells.

For osteogenic differentiation, the cultured cells were incubated in osteogenic medium containing DMEM supplemented with 10% FBS, β-glycerol phosphate 10 mM, dexamethasone 10^-7^M, and ascorbic acid bi-phosphate 50 µg/ml (Sigma). The culture media were changed twice a week for 3 weeks. Afterwards, cells were fixed in 4% paraformaldehyde for 20 min, washed with PBS and stained with Alizarin-Red solution (in 60% isopropanol) for 10 min, followed by repeated washings.

To induce adipocyte differentiation, USSCs were cultured in DMEM supplemented with 10% FBS, dexamethasone 250 nM, insulin 66nM, isobutyl-methylxanthine 0.5 mM, and indomethacine 0.2mM for 3 weeks and also for specific staining, cells were fixed and incubated with Oil-Red. 

***USSCs were cultured as a feeder layer***

These isolated cells were cultured on a 48-well plate. When the cells reached 80% confluence, their mitotic activity was disabled by Mitomycin-C 10 µg/ml (Sigma) according to its protocol: culture media was removed and cells were incubated by mitomycin-C for 2 hours. After incubation time it was washed 2 times by PBS and fresh media was added.

For 3D culture, these cells should seed on the scaffolds. First MBA and DBM slices were cut to the desired size (6 to 8 mm sided cubic pieces) by the sterile scalpel. Then the surfaces of the scaffolds were prepared. To prepare and dispose, they were immersed overnight in the PBS with gelatin 0.1 % which opens the pores like a spongy tissue, ready to work. Afterwards, nearly 3×10^6^ USSC cells from three T75 culture flasks were removed by the trypsin 0.25% (GIBCO). This number of cells in the DMEM media supplemented by 30% FBS were added to MBA and DBM scaffolds (9 pieces each). This concentrated cell suspension was rotating for 8 hours during the incubation. After this time the media was replaced by fresh DMEM. This rotation is needed to push the cells into the pores of the scaffolds. After 3 days, this feeder layer can be disabled by Mitomycin-C too. These scaffolds were placed in 18 wells of a 48 well plate that were previously coated with a USSC feeder layer (9 wells for DBM and 9 wells for MBA).

***Scanning*** ***e*** ***lectron*** ***m*** ***icroscope (SEM)***

For SEM imaging, we should choose a process that preserves the structure of freshly killed material in a state that most closely resembles the structure and/or composition of the original living state. For chemical fixation as the non-coagulative process, we need paraformaldehyde 4%. In this process, first the culture media was removed, and fixed in paraformaldehyde (pH 7) at 4^o^C for 20 min. the scaffold samples were then dehydrated through a graded ethanol series and ultra-structurally scoped using an electron microscope (Philips). 

***HSC isolation by MACS (*** ***m*** ***agnetic*** ***a*** ***ctivated*** ***c*** ***ell*** ***s*** ***orting) method***

MNCs were isolated as described above, and then the CD34+ cells were isolated from this MNC population, according to the manufacturer’s instructions for the CD34 cell isolation kit (Miltenyi Biotech, BergischGladbach, Germany). The cell suspension was adjacent by blocking agent and an antibody labeled with a magnet to one hr at 4°C and dark place. This antibody was against CD34 marker. 

After this period, samples were washed with EDTA-PBS buffer. Afterwards, the suspension was passed through the LS separation columns (Miltenyi Biotech, BergischGladbach, Germany) and eventually with the help of the piston and pressure cells, CD34+ from other cord blood mononuclear cells were separated. As a result the purity of CD34+ cells ranged from 85 to 95%.

***Ex vivo expansion of cord blood hematopoietic cells***

Isolated hematopoietic cells were then cultured in StemSpan (Stemcell Technologies #09600) containing stem cell factor (SCF) 100 ng/mL and FMS-like tyrosine kinase-3 100 ng/ml, and 100 ng/mL thrombopoietin. They were separated and cultured in 48 well plates in four culture conditions: first in 2D culture (without feeder layer and scaffolds) that is simple 2D culture (S2D), second in 2D culture with the USSCs as a feeder layer (F2D), third in 3D culture (DBM scaffold accompanied by USSC feeder layer) and fourth in 3D culture again (MBA scaffold accompanied by USSC feeder layer). In every condition they were cultured in 9 wells of 48-well plates. These 9 wells were counted within 3 weeks expansion and were done triplicate per week.

***Flow cytometry***

Hematopoietic cells were analyzed before and after 3 weeks *ex vivo* expansion by two-color ﬂow cytometry (PARTEC, Germany). Nearly 10^5^ cells were re-suspended in PBS-5% FBS and were stained with ﬂuorescein isothiocyanate (FITC)-conjugated anti-human CD34. These cells were gated with low side scatter. In order to confirm the speciﬁcity of staining, we stained hematopoietic cells by an isotype control with FITC-mouse IgG1 in a replicate manner to ensure the speciﬁcity of staining. Acquired data using PARTEC ﬂow cytometer were then analyzed by FloMax software.

***Clonogenic assay***

In the isolation day and at the end of 1^st^, 2^nd^ and 3^rd^ weeks for all 3 groups, we prepared three dilutions of expanded cells 500, 1000, 1500 per each culture condition. Every cell dilution was mixed with 1 ml of Methocult™ GF+ H4435 methylcellulose medium (Stemcell Technologies #04435) in separate tubes, according to the manufacturer’s instructions, the contents of each tube were plated within separate wells of 6-well plates. Plates were incubated at 37°C with more than 95% humidity and 5% CO_2_ for 2 weeks. To maintain culture humidity, one well was filled with sterile water.

**Figure 1:**
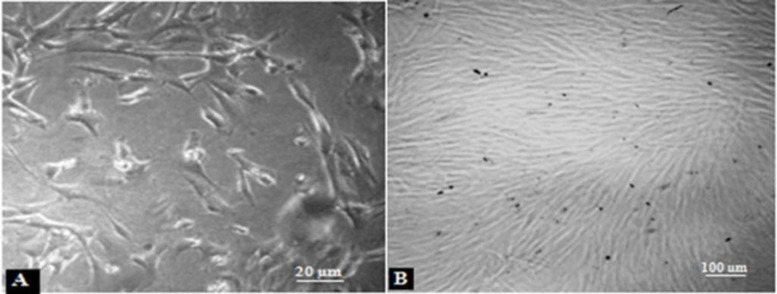
USSC cells in the specific media. **A**) USSC colonies after 2 weeks. **B**) USSC cells by 80% confluency

***Long-term culture-initiating cells*** ***(*** ***LTC-IC*** ***)***

LTC-IC assay was performed according to the technique described below*.* Bone marrow cells were cultivated as feeder-layer cells in 96-well plate format. These cells were harvested and incubated with mitomycin-C. A day later, the stromal layers were charged with CD34+ cells in the specific Myelocult medium (Stemcell Technologies #05100) containing 10^−6^M hydrocortisone (Stem Cell Technologies *#*07904). A minimum of 1  l0^3^ up to a maximum of 4 × l0^3 ^expanded hematopoietic cells per well were plated. After 5 weeks of incubation, cells were removed and assay in the CFC assay as described above. A well is scored as positive if one or more BFU-E, CFU-GM or CFU-GEMM is detected or as negative if no colonies are present. The LTC-IC frequency in the test cell population is calculated from the proportion of negative wells (no CFC present) and the method of maximum likelihood then statistical analysis can be performed.

***Mitotic chromosome preparations for karyotyping***

After high expansion in cell count, the cells should be checked for chromosome number and banding pattern to determine if they were altered from the normal (expected) pattern. For harvesting cells, at first 0.15 ml of 50 µg/ml Colchicine solution was added to each culture tube containing expanded CD34+ cells and followed by incubation at 37°C and centrifuge. Then 2-3 ml of prewarmed 0.075 M KCl solution was added to the cell pellet which after 10 min centrifugation at 500×g, were gently pipeted by fixative solution (3:1 (v/v) methanol/glacial acetic acid) followed by incubation at room temperature and centrifuge. This step was repeated twice more by applying a small drop of cell suspension onto the surface of a slide and allowing it to spread. Slide was stained by Giemsa solution.

***Statistical analysis***

Results were expressed as mean ± SD and analyzed by the T test. Differences were considered to be significant at *P*< 0.05. 

**Figure 2:**
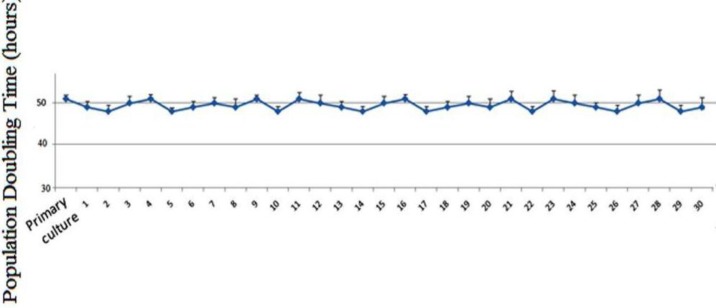
Population doubling time of USSCs cells in primary culture up to the 30 passages. Average of PDT was 49.48 ± 0.3 hr

## Results

***Cell culture***

USSC colonies were observed after 2 weeks of USSC cell culture (Figure 1A). After each passage these spindle-shaped cells reached complete confluency (Figure 1B). It didn’t take longer to reach confluency and their morphology didn’t change with each passage. The results related to the PDT (population doubling time) in different passages of the USSCs are shown in Figure 2. PDT didn’t change during the experiment periods from primary culture to passage 30.

***Characterization of USSCs by flow cytometry***

**Figure 3:**
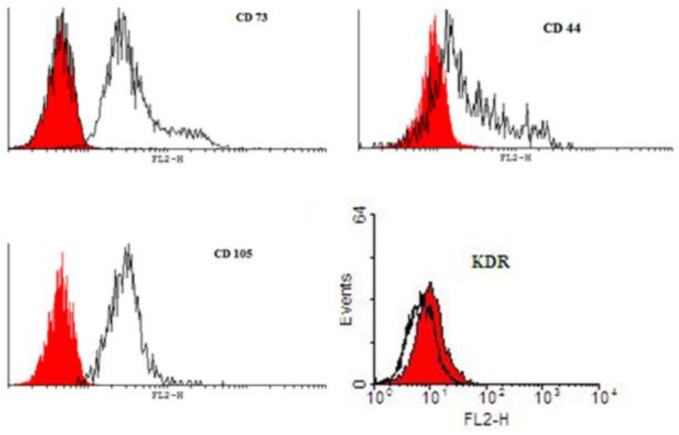
Immunophenotypes of USSCs. USSC preparations were labeled with the monoclonal Abs specific for the molecule indicated (filled histograms) or isotype controls (open histograms). USSC are positive for CD73, CD44 and CD105 but have weak positive expression for KDR

USSCs in 4^th^ passage were subjected to flow cytometry for the presence or absence of hematopoietic and endothelial markers. USSCs typically expressed the surface antigens CD105, CD44 and CD73 (Figure 3), and showed low level of KDR but were negative for CD34 and CD45, which are the early hematopoietic markers. Analysis of cell surface markers by FACS analysis were confirmed these isolated cells.

***Differentiation of USSCs to*** ***osteogenic and adipogenic***

**Figure 4:**
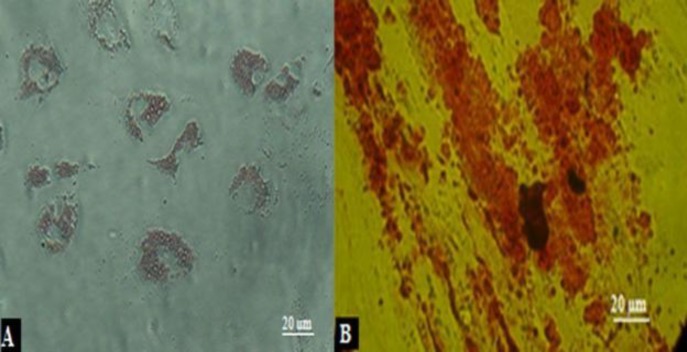
Isolation and differentiation characteristics of human UCB-derived USSCs. **A) **The results of Oil Red-O-staining in differentiated adipogenic cells. **B) **Results of osteogenic differentiation by Alizarin Red-staining.

USSC cultures in adipogenic differentiation medium after 2 weeks, presenting fat vacuoles in the cytoplasm can be appeared by oil red staining (Figure 4A). For osteogenic inductive medium, appearance of refringent crystals were be observed by Alizarin Red staining (Figure 4B).

***Scanning electron microscope views***

**Figure 5:**
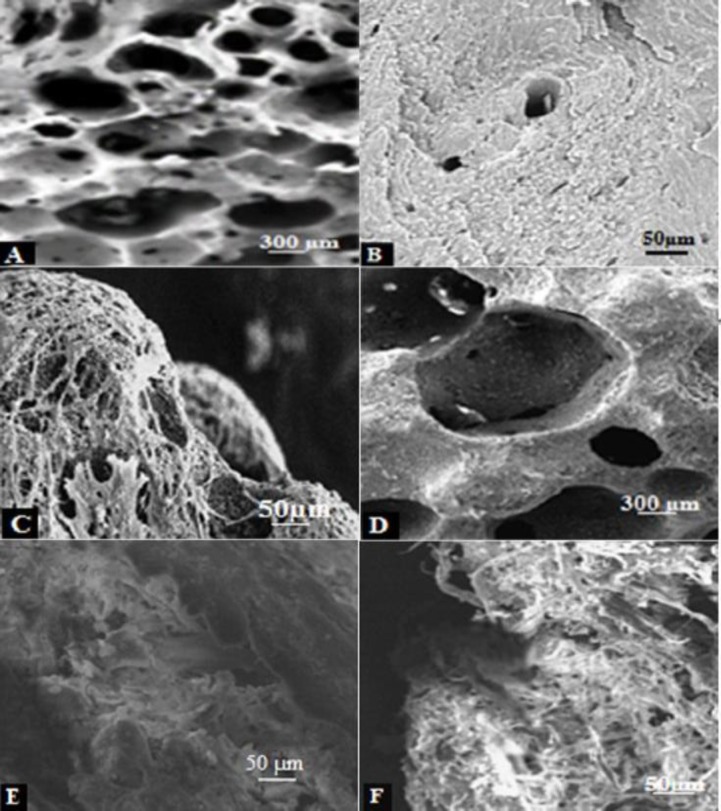
SEM views of scaffold surface in black and white version. **A-B)** empty MBA without USSC cells. **C) **MBA with seeded USSC. **D-E) **empty DBM without USSC cells. **F)** DBM with seeded USSC

About 40% of scaffold surface pictures showed porosity. Figure 5 shows the surface of scaffolds without cells as the control, so that very small pores in the scaffold can be seen under a microscope in which these pores are so deeply dark. When the scaffolds were seeded by USSCs as the feeder layer, the empty scaffold is less clear. Elongated and spindle-shaped cells covered the surface but few of these surrounded cells entered into the cavities and pores. Cells were more coated on the outer face. Cell size was about 100 µm and with more magnification each cell could be observed separately as a light band. However, with the same zoom level on the control scaffold, only the dark face without the light bands were visible (Figure 5B, E).

**Table 1:** The effect of USSC and scaffold combination on the expansion of UCB-CD34+ over 3 weeks. Values are expressed as mean ± SD for 3 numbers of experiments. Fold expansion was calculated based on the cell counts (1000) on day 0

Total Cells (fold exp.)			
Groups	1^st^ wk	2^nd^ wk	3^rd^ wk
S2D (n=3)	13800±1600 (13.8±1.6)	53400±3200 (53.4±3.2)	101900±8000 (101.9±8)
F2D (n=3)	28700±3400 (28.7±3.4)	104300±11000 (104.3±11)	194200±4600 (194.2±4.6)
MBA (n=3)	40800±4100 (40.8±4.1)	145500±5800 (145.5±5.8)	271700±13000 (271.7±13.2)
DBM (n=3)	44200±1600 (44.2±1.6)	176700±6400 (176.7±6.4)	305800±12700 (305.8±12.7)

Abbreviation: simple 2D culture (S2D), simple 2D culture with feeder (F2D), mineralized bone allograft (MBA), de-mineralized bone matrix (DBM)

***Ex vivo*** ***expansion of cord blood hematopoietic cells***

**Figure 6:**
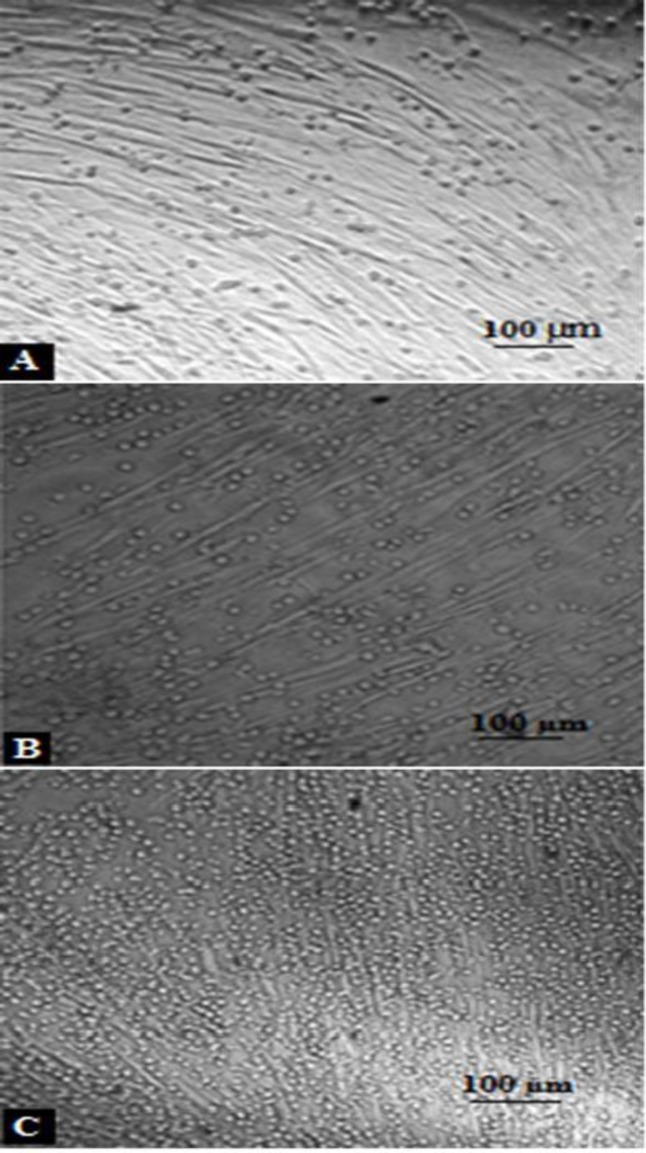
USSCs co-cultivate with UCB-CD34+ over 1 **A)**, 2 **B)**, and 3 **C)** weeks expansion

UCB CD34+ cells were seeded at a concentration of 1×10^3^ cells per each well in the 48-well plate in the hematopoietic stem cell expansion medium (StemSpan supplemented by 3 cocktail cytokines: Flt3-L, SCF, TPO). CD34+ cells in 4 groups were expanded: S2D, F2D, DBM and MBA. These 4 groups were counted after each week during 3 weeks by triplicate for each group. The result is shown in Table 1. In F2D culture, the CD34+ cells were co-cultivated on the USSC-feeder layer. USSCs showed their ability to support the expansion of CD34+ cells over three weeks (Figure 6). The highest cell count was in 3D culture but DBM was a little more than MBA. The total cell count on the USSC layer in DBM culture increased within the 21-day period, it was 44.2-fold ± 1.6-fold after 7 days, 176.7-fold ± 6.4-fold after 14 days, and 305.8-fold ± 12.7-fold after 21 days.

**Table 2:** The effect of USSC presence as the feeder layer on the colony formation of UCB-CD34+ over 3 weeks. CFC counts were performed from the expanding cultures at days 0, 7, 14, and 21. The results are expressed as mean ± SD for total cells, CFC, BFU-E/CFU-E, CFU-GM, and CFU-mix

	Total cells	CFC /10^3 ^cells	BFU/CFU-E /10^3 ^cells	CFU-GM /10^3 ^cells	CFU-mix /10^3 ^cells
Day 0	1000	30.9±2.1	22.6±0.7	9.5±1.1	2.8±0.3
1^st^ week					
F2D	28700	23.2±1.5	9.4±0.5	12.2±0.7	1.6±0.3
MBA	40800	16.0±1.8	6.1±0.3	7.3±1.1	1.0±0.4
DBM	44200	16.3±2.7	7.3±0.9	7.9±1.7	1.1±0.1
2^nd^ week					
F2D	104300	35.6±1.8	8.8±1.2	26.2±0.6	0.6±0.0
MBA	145500	28.6±2.0	7.1±0.7	20.7±1.3	0.8±0.0
DBM	176700	30.1±3.3	7.9±2.1	21.9±0.8	1.9±0.4
3^rd^ week					
F2D	194200	24.4±1.8	6.2±0.9	17.1±0.7	1.1±0.2
MBA	271700	22.5±1.4	3.1±0.6	17.9±0.5	1.5±0.3
DBM	305800	23.9±1.7	4.7±0.3	18.4±1.0	1.0±0.4

Abbreviation: simple 2D culture with feeder (F2D), mineralized bone allograft (MBA), de-mineralized bone matrix (DBM), Colony Forming Count (CFC), Burst Forming Unit/Colony Forming Unit- Erythroid (BFU/CFU-E), Colony Forming Unit- Granulocyte/Macrophage (CFU-GM), Colony Forming Unit- mix (CFU-mix)

***Flow cytometry***

**Figure 7:**
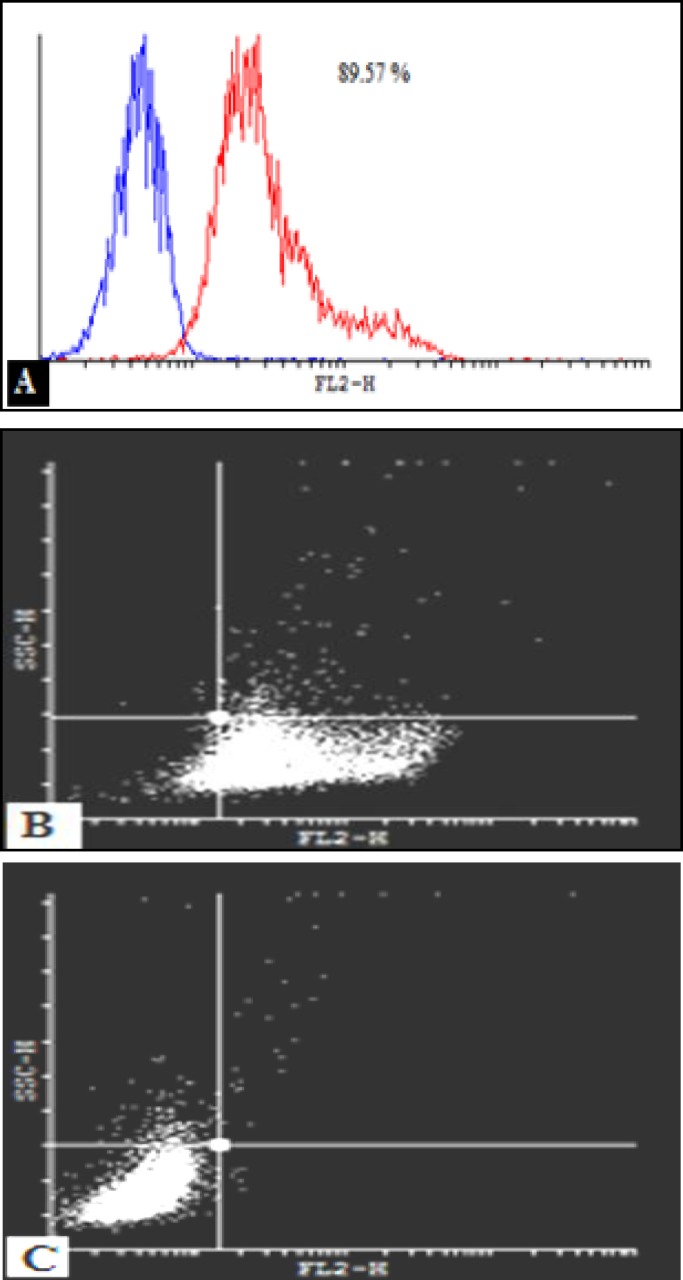
**A)** enumeration of CD34+ at isolation day was 89.57 percentage.** B)** CD34+ cells were labeled with FITC-conjugated antibody and **B)** with IgG1 as the control antibody

**Figure 8:**
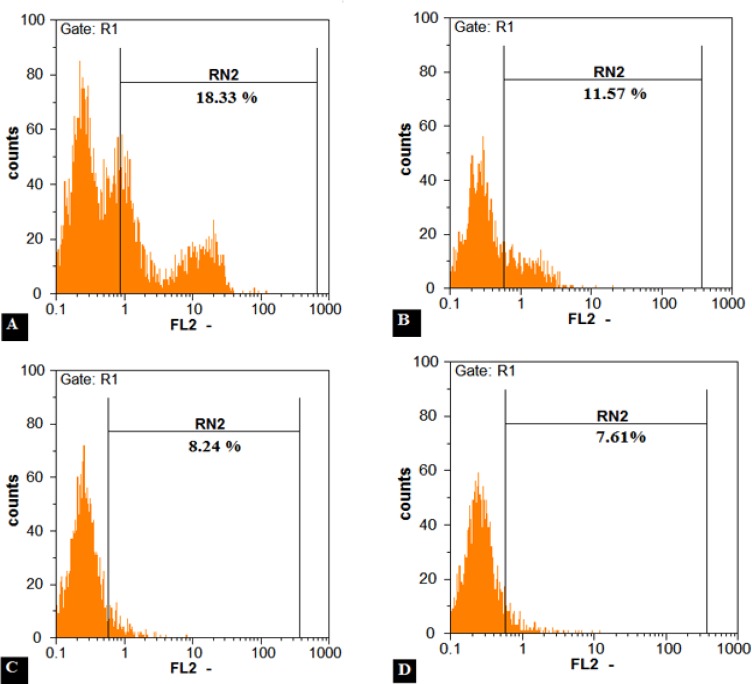
The CD34+ stem cell populations after 3 weeks *ex vivo* expansion were evaluated by means of flow cytometry. Flow cytometry assay showed that 18.3 % for S2D **A)**, 11.5 % for F2D **B)**, 8.2 % for MBA **C)** and 7.6 % for DBM **D)**

CD34+ cells were enriched by nearly 90 percent (Figure 7) and subsequently after 3 weeks expansion, CD34+ cells were analyzed by FACS method to observe their CD34 marker ratio (Figure 8). The global competition indicated that the lowest CD34 marker belonged to the 3D culture (8.6 % and 9.2% respectively for DBM and MBA). But no significant difference was observed between MBA and DBM in the kind of 3D condition. The highest CD34 marker was observed in S2D culture (17.3%) and finally for the F2D group it was 10.5 %. Percent drop in CD34+ cells was greater in the three-dimensional culture. This problem can partly explain the high levels of secreted factors attributed myeloid line distinction, such as GM-CSF, M-CSF, IL-6, IL-11 and SCF. Also the IL-1B factor secretion by both USSC and CD34+ cells strongly enhanced the G-CSF production by USSC cells leading to the differentiation of CD34+ cells into a specific cell line. This low percentage in contrast to the highest expansion and CD34+ count in 3D condition can be ignored.

**Figure 9:**
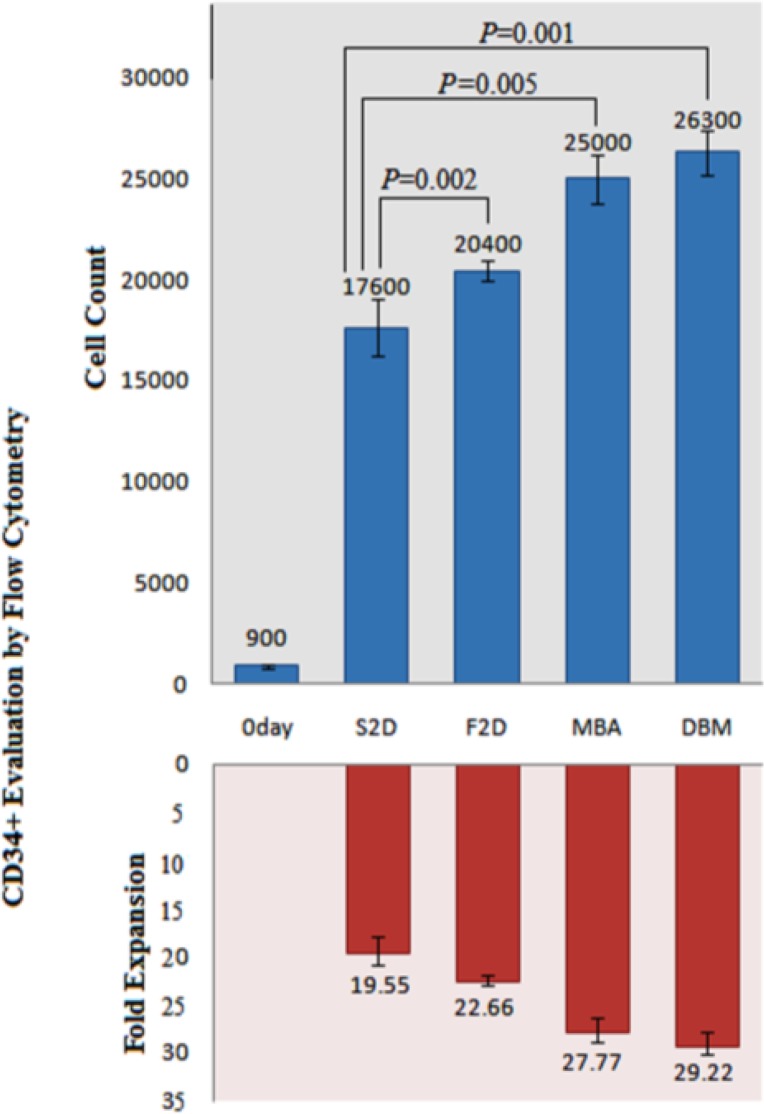
Kinetics of *ex vivo* expansion of CD34+ cells by flow cytometry in different culture conditions after 21 days. The results are expressed as mean fold ± SD for S2D, F2D, MBA and DBM.      *P-value *compare to the S2D for each condition was significant

On the first day we charged each group by 1000 cells with 89.57% CD34+ cells or nearly 900 CD34+ cells. For S2D, we counted 101900 cells by 17.3% or about 17600 CD34+ cells. Therefore fold expansion by flow cytometry was 19.55-fold ± 1.5-fold. Other HSCs expansion evaluation by flow cytometry is shown in Figure 9. The most expansion belonged to the DBM group.

***Colony assay***

**Figure 10:**
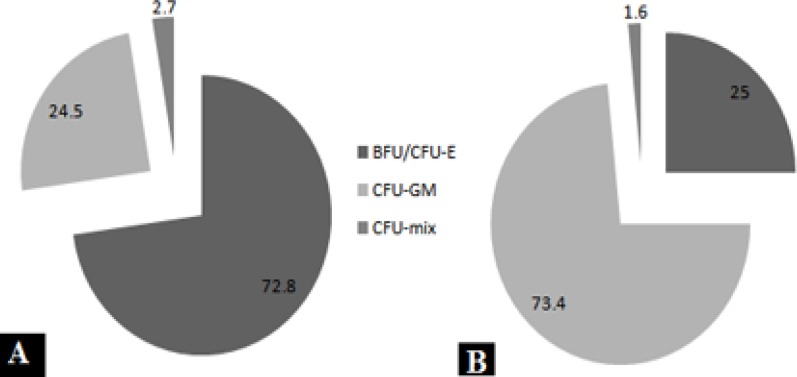
Kinetics of colony formation of CB-CD34+ cells expanded on USSC. CFC counts were performed from the expanding cultures at days 0 **A)** and 14 **B)**

Each week, nonadherent hematopoietic cells (CD34+ cells) from the adherent feeder cells were separated and analyzed by CFC to evaluate the progenitor content and CD34+ cells. As summarized in Table 2 and Figure 10. For total CFC, the highest CFC expansion was observed at day 14: 35.6-fold ± 1.8-fold, 28.6-fold ± 2.0-fold and 30.1-fold ± 3.3-fold for F2D, MBA and DBM, respectively. Similarly, amplification of CFU-GM and BFU-E/CFU-E was highest, whereas CFC was reduced after 2 weeks. The composition of CFC clearly showed that after 2 weeks about 73% of colonies obtained were CFU-GM. But on day 0 nearly 73% of colonies were BFU-E/CFU-E. When total number of cells generated in 3D culture was compared to 2D culture, significantly higher colony counts for 3D were determined at each time point. Because these colony counts were in 1000 cells, so that in comparison with total cells in F2D, MBA and DBM the CFC counts were 3713±188, 4161±291 and 5318±583.

**Table 3:** The frequency of long-term culture-initiating cells in different condition during 3 weeks expansion

Frequency of LTC-IC cells					
Day 0	1 in 8372				
1^st^ wk		2^nd^ wk		3^rd^ wk	
S2D	1 in 11634	S2D	1 in 14840	S2D	1 in 22478
F2D	1 in 7389	F2D	1 in 5125	F2D	1 in 11650
MBA	1 in 7760	MBA	1 in 6192	MBA	1 in 13289
DBM	1 in 7554	DBM	1 in 5992	DBM	1 in 12673

Abbreviation: simple 2D culture (S2D), simple 2D culture with feeder (F2D), mineralized bone allograft (MBA), de-mineralized bone matrix (DBM)

***LTC-IC***

After 3 weeks co-cultivation of CD34+ cells on the USSC layer (2D and 3D conditions) no significant differentiation between them was observed in contrast to the simple 2D culture where the total number of long-term culture-initiating cells dropped after each week (Table 3). The culture by feeder (F2D, MBA and DBM) could make an expansion in 1.13-fold, 1.07-fold and 1.10-fold in the first week and also in 1.63-fold, 1.35-fold and 1.39-fold in the second week in contrast to the day 0. But in the 3^rd^ week this expansion dropped and maintaining of LTC-IC cells reached nearly 0.6-fold. Therefore USSC cells could only preserve the hematopoietic initiating cells just for 2 weeks in the *ex vivo* condition and make long term hematopoesis. 

***Karyotyping***

**Figure 11:**
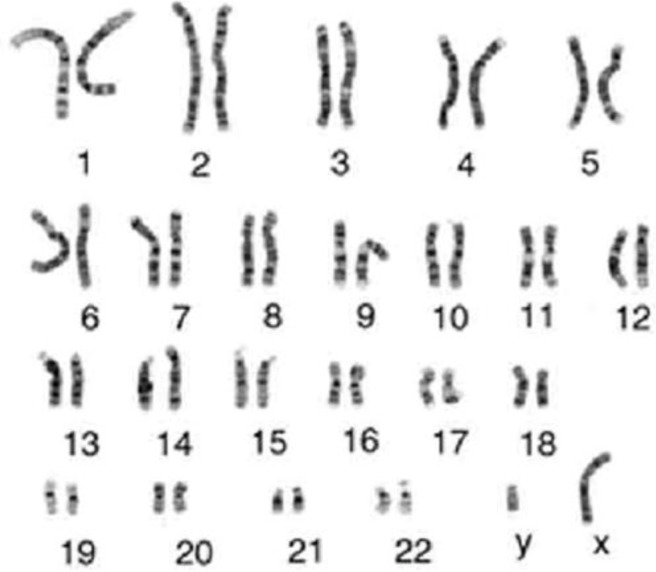
Karyotype of the expanded CD34+ cells after 21 days in 3D condition. Chromosome number is 46 like a normal cell and no fusion between stromal and HSCs in cytogenetic term were obtained

For research in genetics and cytogenetics, Karyotyping was developed. The G-banded karyotype is useful in the identification of aberrant chromosomes and absent, additional, or aberrant chromosomes, which can include deletions, inversions, reciprocal translocations, duplications, insertions, and Robertsonian chromosomes. Figure 11 shows the G banding pattern in normal chromosomes. No aberrations were obtained and did not alter the normal banding pattern. The human karyotype contains 22 autosomal pairs and two sex chromosomes. The female karyotypes contain two large X chromosomes (the inactive X may be very darkly stained), whereas the male karyotypes contain one large X chromosome and a small one. Normal karyotype of the expanded CD34+ cells after 21 days in 3D DBM condition by 305.8-fold expansion was obtained. Chromosome count was 46 as in normal cells and no abnormalities in quantity (aneuploidy) or quality were observed and no fusion between stromal and HSCs occurred. 

## Discussion

In previous studies, at first, nutritious feeder layer (mesenchymal stem cells) was used for cell proliferation as the Dexter culture (1977). But Miller showed that growth factors can also create a similar environment for expansion (22). The nutrients and growth factors layer, for cell proliferation of SCID/ NOD were used simultaneously (23). But they also understood that over a long period of time, this environment cannot provide reliable cell (24) and showed that stem cells are sensitive to their microenvironment. Here the porous tantalum scaffold (tantalum-coated porous biomaterial (TCPB)), only after a week, showed 1.5 increasing fold compared with the two-dimensional culture (25). In this regard, the engineering matrices and other polymer or nanomer materials were used (26-31). The result of these experiments for replication (32-34) or differentiation (35) of stem cells was performed. Finally it caused better BMT and other hematic transplantations (36-38) or even without entering the clinical stage, biological or synthetic structures were used for stem cell proliferation (39-42). In Tan and colleagues research -2011(43), three-dimensional structure was derived from the bone tissue for stem cell proliferation to achieve 7.5-fold expansion. In this structure a feeder layer of mesenchymal stem cells was used but scaffolding explanation was not provided so the real significance of this scaffold is the substances that are present. In other studies, non-mineralized bone matrix was used as scaffold. With general comparison of previous research, we want to investigate the applicability and effectiveness of simultaneously using the mineralized scaffold with USSCs feeder layer for proliferation of hematopoietic stem cells (15, 17, 44-47). The results of our research are also in line with previous studies and showed that proliferation in three-dimensional conditions is better.

b

In order to improve efficiency of the tissue and cell culture in tissue engineering, we need to create three-dimensional *ex vivo* conditions that mimic inside of body (*in vivo*). For this purpose, the cells are cultured on the suitable scaffolds. To achieve this goal, we need to mimic the inside of body which contains co-culture of purpose cells with the stromal cells simultaneously (51, 52). MNCs cannot form colonies in the culture without supportive environment (53).

As described above the only difference between DBM and MBA is the lack of mineral in DBM. We know that bone marrow is composed of a protein matrix and a mineral section necessary for hardness, rigidity and stability that enables the skeleton to resist gravitational and mechanical loading, also has the ability to transfer specific signals to stimulate cell division of stem cells. Features of these two scaffolds are similar to bone tissue and were used for bone tissue engineering (54). 

Three-dimensional geometry and topography of these porous scaffolds with the adherent stromal cells (USSCs) could emulate the bone marrow microenvironment and hematopoesis (55, 56). For HSCs, sticky stromal cells are considered as the co-culture to increase the density of blood cells (57, 58). Production of extracellular matrix, hematopoietic cytokine, connection and interaction between molecules could be increased in the small cell area. Therefore DBM and MBA by USSC can be a suitable *ex vivo* mimicry niche by intensifying surface/volume ratio (59) and supporting the stem cell differentiation and expansion.

Finally, the production of nutrient materials, growth factors, hormones, autocrine, paracrine, and exocrine (60) factors will be maximum just in the small location called local concentrations of factors (61). Stromal cells are a source of adhesion molecules and growth factors for supporting stem cell expansion (62). 

USSCs in contrast to the mesenchymal stem cells have the longer telomerase that cause more expansion capacity and self-renewal in these cells. USSC can produce a lot of cytokines such as CSF, LIF (Leukemia Inhibitory Factor), TGF-1β (Transforming Growth Factor-1 beta), VEGF (Vascular Endothelial Growth Factor), GM-CSF, M-CSF, IL-1β, IL-6, IL-8, IL-11, IL-12, IL-15, SDF-1α (Stromal Derived Factor-1 alpha) (56). 

When feeder cells are in contact with the environment in three dimensions, the result is increased secretion of factors that can improve the efficiency of the feeder. Therefore focus on three factors increased secretion and this effect is direct contact of the humeral (61). The scaffold was a 6-8mm sided cube and porosity was 40% so every aspect of the six-way area scaffold was 68.6mm^2^ {(7×7)+0.4×(7×7)}. With regard to the sixth aspect, the general area of ​​the scaffold was 411.6mm^2^. The well’s diagonal of each 48-well plate was 12mm therefore well area was 113.04 mm^2^. Finally for MBA and DBM the general surface was 524.64mm^2^ that was 4.64-fold of S2D.

## Conclusion

Stem cells in the body depends on the three-dimensional mode will be simulate to differentiate or prolife state. Their power of proliferation cannot survive for long outside of niche (63). Niche could control the number of stem cells in the body and prevent them from multiplying too (12).

Stem cell expansion out of the niche such as in 2D culture or even in the erythropoesis Dexter culture with abnormal face, or uncontrolled proliferation can lead to aneuploidy and cancer.

HSCs niche has helper cells with basic materials. This background material or extracellular matrix has a lot of proteins such as fibronectin (64), collagen (65-66) and gelatin (67) that were synthesized for a lot of scaffolds based on them (68). Acid extraction removes the minerals thus the protein surface of the scaffold (flexible microenvironment) is presented which is familiar for the cells. Therefore DBM will be so soft, porous and spongy (45) and the rate of cytokine production and *ex vivo* hematopoiesis-supporting activity of UCB- derived USSC in 3D culture is improved.

This is therefore suggested for better 3D culture results in tissue engineering. This result shows that multiline age expansion of CD34 derived UCB is feasible in the feeder contact cultures without further addition of cytokines.
